# Sociodemographic and Clinical Characteristics Associated with Improvements in Quality of Life for Participants with Opioid Use Disorder

**DOI:** 10.3390/healthcare10010167

**Published:** 2022-01-16

**Authors:** Assaf Gottlieb, Christine Bakos-Block, James R. Langabeer, Tiffany Champagne-Langabeer

**Affiliations:** 1 School of Biomedical Informatics, University of Texas Health Science Center at Houston, 7000 Fannin St., Houston, TX 77030, USA; Assaf.Gottlieb@uth.tmc.edu (A.G.); christine.bakosblock@uth.tmc.edu (C.B.-B.); james.r.langabeer@uth.tmc.edu (J.R.L.); 2 McGovern Medical School, University of Texas Health Science Center at Houston, 6431 Fannin St., Houston, TX 77030, USA

**Keywords:** quality of life, opioid use disorder, counseling, relapse, treatment

## Abstract

Background: The Houston Emergency Opioid Engagement System was established to create an access pathway into long-term recovery for individuals with opioid use disorder. The program determines effectiveness across multiple dimensions, one of which is by measuring the participant’s reported quality of life (QoL) at the beginning of the program and at successive intervals. Methods: A visual analog scale was used to measure the change in QoL among participants after joining the program. We then identified sociodemographic and clinical characteristics associated with changes in QoL. Results: 71% of the participants (*n* = 494) experienced an increase in their QoL scores, with an average improvement of 15.8 ± 29 points out of a hundred. We identified 10 factors associated with a significant change in QoL. Participants who relapsed during treatment experienced minor increases in QoL, and participants who attended professional counseling experienced the largest increases in QoL compared with those who did not. Conclusions: Insight into significant factors associated with increases in QoL may inform programs on areas of focus. The inclusion of counseling and other services that address factors such as psychological distress were found to increase participants’ QoL and success in recovery.

## 1. Introduction

Quality of life (QoL), according to the World Health Organization, is a “state of complete physical, mental, and social well-being”, is achieved not simply by the absence of disease, but is largely subjective to change according to individual perceptions, experiences, and expectations [1]. While chronic and acute illness significantly affects the overall quality of a participant’s life, including physical health, mental, and social wellbeing, it also affects relationships and meaningful participation in social activities [2]. Because mental and social wellbeing are essential for overall health, the model for chronic disease management includes long-term medical, behavioral, and social care that is coordinated, if not integrated. Chronic disease management incorporates mental health and social support with medical care, and has been shown to improve participant outcomes and QoL [3,4]. Substance use disorder is acknowledged as a chronic illness that negatively impairs the quality of a patient’s life, and they therefore would benefit from the application of the chronic disease model of integrated medical, behavioral, and social support [5,6]. The recommended course of treatment for opioid use disorder (OUD) is medication and behavioral health counseling; and although not always included, social support services are vital to improving QoL [7,8,9]. Social support has been shown to positively influence treatment initiation and retention [8]. Perceived social support may significantly affect improvements in different domains of QoL, including mental and physical health, and social wellbeing [8].

Quality of life is multidimensional, dynamic, and consists of objective and subjective measures. Objective measures can be quantified, (e.g., time in treatment, abstinence, housing status, age, and gender); however, the relationship between objective and subjective QoL may be harder to determine. Although relatively small, the consensus of research on QoL among individuals with OUD substantiates QoL as an important factor in treatment and recovery, and it is not a simple linear correlation [10,11,12,13,14,15,16,17,18,19]. It is widely accepted that QoL is more than simply physical health. To a large extent, psychological and physical wellbeing are mutually dependent. For instance, the reciprocal relationship between psychological pain and physical pain has been established [20]. Additionally, many QoL assessments that are disease-specific also include measures for social and psychological domains [21,22]. Psychological distress is a strong predictor of poor QoL, independent of physical and social factors [23,24]. Moreover, psychological distress is a primary factor in vulnerability to addiction and in treatment retention and relapse [25,26]. Although QoL is considered crucial to sustained recovery from OUD, there are no measurements specifically geared toward this population, and research focusing solely on QoL among those with OUD receiving buprenorphine is limited and sometimes contradictory [21,27]. In a recent review of QoL changes among participants with OUD receiving medication for opioid use disorder (MOUD), a few studies showed an initial increase from baseline, then diminishing and even declining rates [13,17,18,28]. Other studies have found that methadone treatment produced initial improvements in QoL; however, other factors, such as age of greater than 50 years and a positive human immunodeficiency virus (HIV) status had a negative effect [29]. Still, a few studies found rates of QoL increased and did not report decreases [30,31,32]. Even with slight differences in results, the research supports that QoL is lower among people with OUD than with the general population [27,33] and people with other chronic illnesses [34]. This research examined multiple sociodemographic and clinical characteristics of participants within a treatment program for OUD to determine factors associated with improvements in QoL over time.

## 2. Materials and Methods

### 2.1. Setting

The Houston Emergency Opioid Engagement System (HEROES) is an inter-agency, emergency opioid treatment program that encompasses hospital and community-based emergency medical services (EMS), certified peer recovery support services, behavioral health services, family support, and expedited access to MOUD to high-risk individuals who have recently survived an opioid overdose [35]. The primary mode of entry into this program is through EMS referral and assertive outreach within 72-h of an overdose event. Using 911-call data, assertive outreach occurs through a two-person team consisting of a licensed paramedic and a licensed peer recovery support specialist. The team visits the home of the participant after a recent overdose, with the purpose of engaging them into a treatment program. The program is staffed with nurse practitioners who provide MOUD, licensed chemical dependency counselors who provide substance abuse counseling, peer recovery support specialists who provide support groups and regular check-ins, and a social worker and navigators who provide additional resources when needed. The preliminary results from this program indicate approximately 88% of participants are retained at 30-days post enrollment [36].

This study took place in the Houston metropolitan service area, with a population 5.9 million people, in a large academic medical center [37]. Participants in this study were enrolled in the Houston Emergency Opioid Engagement System (HEROES), which is based at the University of Texas Health Science Center at Houston and is registered as a national clinical trial (NCT 03396276).

### 2.2. Measures

Quality of life was measured using the EuroQol-Visual Analog Scale (EQ-VAS), where participants are asked to rate how they view their overall health at the time of assessment, from zero (worst imagined health) to 100 (best imagined health) [38]. We considered baseline as the QoL score of the first assessment, as long as it was within 30 days of joining the program. We tested the association of 145 factors collected on participants with changes in QoL (both negative and positive changes) from baseline using Pearson correlation for continuous factors and Mann−Whitney U test for dichotomous factors. The factors include sociodemographic and clinical factors such as gender, race, age, socioeconomic status like housing, transportation, familial status, insurance type, history of prescription and illicit drugs, vitals, mental health, and past legal problems. All data used for this research were maintained in a REDCap database and were analyzed using Matlab version R2021a [39].

### 2.3. Participants

This study was a retrospective analysis of the data collected from intake and follow-up assessments of patients enrolled from 24 January 2020 to 24 April 2021. All services provided by HEROES were provided to patients at no charge. Participants provided written informed consent to participate in research prior to data collection, and this study was approved by the Committee for the Protection of Human Subjects at the University of Texas Health Science Center at Houston.

## 3. Results

We identified 494 participants that had a baseline QoL measurement (defined as a measurement up to 30 days from joining the program) and at least one additional QoL measurement in the program.

**Figure 1:**
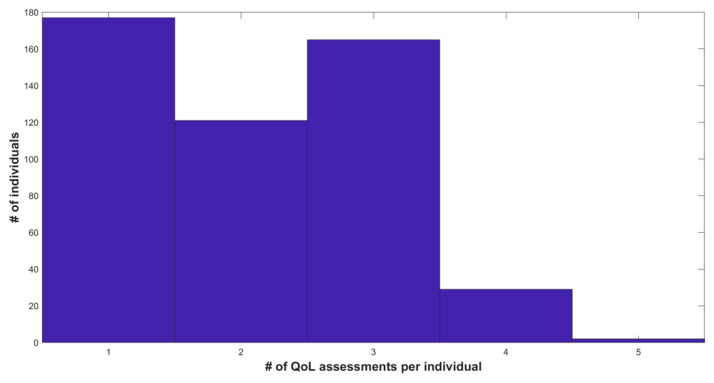
Histogram of the number of QoL assessments conducted per individual after the baseline assessment. #, Number.

**Figure 2:**
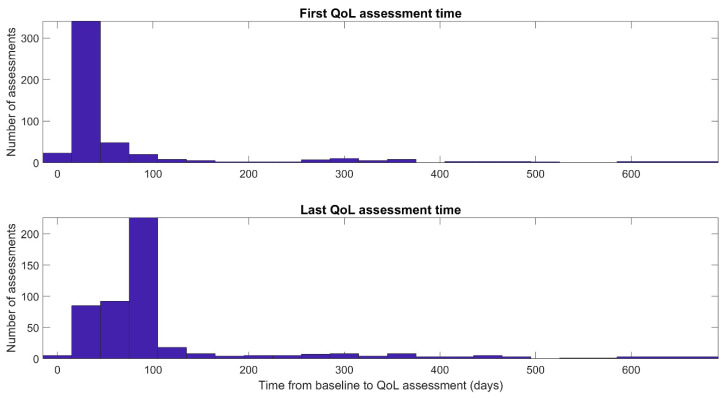
Histogram of the first (**Top**) and last (**Bottom**) time for QoL assessment in days from baseline.

The majority of these participants had between one to three QoL measurements (Figure 1), where the last QoL measurement was conducted on average at 102.9 ± 102.7 days after the baseline, where the bulk of the first QoL assessments were done 30 days after baseline and the last one after 90 days from baseline (Figure 2). There is negligible correlation between the change in QoL and the time that passed between baseline and assessment dates (Pearson ρ = 0.03).

**Figure 3:**
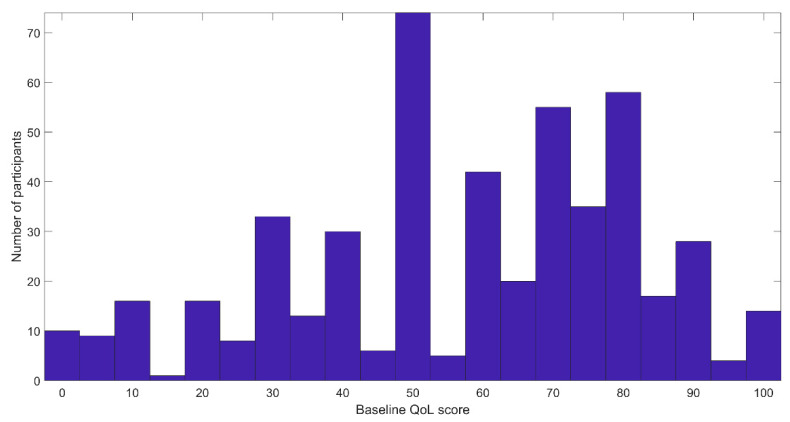
Histogram of baseline QoL scores.

The baseline QoL scores were 58 ± 24.5 (Figure 3).

The demographic characteristics of the participants are listed in Table 1.

**Figure 4:**
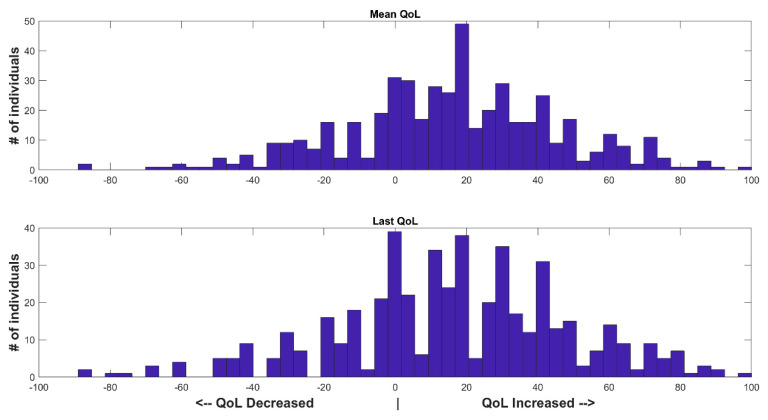
Histogram of the average (**Top**) and last (**Bottom**) change in QoL scores from baseline.

Of the 494 participants, 350 participants (71%) had improved QoL from baseline (Figure 4). The average improvement across all participants was 15.8 ± 29, while the improvement in the last measured QoL of each participant was 16.4 ± 32.3.

We identified 10 factors that passed the Benjamini−Hochberg false discovery rate of 0.05 for association with the QoL score [40].

The top significant factors associated with change in QoL were for participants who reported they had not relapsed since entering the program (7.5 points increase for those who relapsed vs. 22.1 for those who did not; FDR-adjusted *p* < e^−5^). Notably, we did not incorporate whether the participant had relapsed prior to joining the program. The second top factor was whether the participant reported attending sessions with a Licensed Chemical Dependency Counselor (LCDC) and/or a professional counselor (19 points increase for those who attended counseling vs. only 5.5 for those who did not; FDR-adjusted *p* < 0.001) (Table 2).

The other factors associated with reported increases in QoL were a history of polysubstance use (nicotine/tobacco, marijuana, benzodiazepines, alcohol, and non-Rx opiates). Additionally, participants with past legal problems, arrests for shoplifting or vandalism, and past traumatic experiences noted a higher increase in QoL.

**Figure 5:**
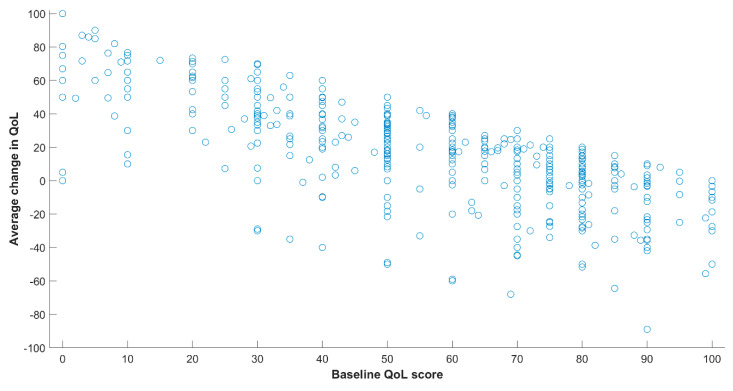
Scatter plot of the change in QoL scores relative to baseline scores.

We identified a negative correlation between the baseline QoL and the average change in QoL (Pearson ρ = −0.7, *p* < 8 × 10^−74^, see Figure 5). We further tested the differences between participants with low initial QoL scores (216 participants with baseline QoL score lower or equal to 50,) and those with high initial QoL (baseline QoL score higher than 50, 278 participants). Figure 3 displays the distribution of baseline scores.

Interestingly, only one factor, namely relapse since entering the program, remained significantly associated with QoL change in the group with an initial low QoL with a similar difference to the overall group (average improvement of 42.8 points for those who did not have a relapse vs. 26.8 for those did relapse). Conversely, in the group with an initial high QoL, 19 factors were associated with QoL change. These 19 factors included the ten factors identified in the entire population, while the additional nine factors included past usage of any of six illicit drugs (illicit opioids, heroin, cocaine, hallucinogens, and amphetamines), two types of prescription opioids, being arrested for assault, and employment as a main source of income. The participants who had used heroin experienced an increase in QoL while in the program, while those who had not experienced a negligible change. Finally, participants with their primary source of income from employment suffered a mild (2 points) reduction in QoL, while those whose source of income was different did not see much change.

## 4. Discussions

We evaluated changes in QoL, and sociodemographic and clinical factors associated with the change at various intervals in an OUD treatment program. This study supports previous research that shows QoL improvements for participants with OUD after entering treatment with MOUD. Participants in this study had a notable increase in their subjective QoL scores from baseline after joining the program. Because this is a short-term program intended to serve as a bridge to long-term treatment, there were no long-term assessments on QoL. Prior research has shown improvements in QoL among participants 6 to 12 months after entering treatment, with declining QoL after 12 months [13,14,17,18,31]. Although an improved QoL can reinforce abstinence, abstinence does not necessarily reinforce a higher QoL; in fact, a low QoL can forecast relapse [12,31]. Summarily, QoL is essential to lasting recovery and therefore the factors associated with QoL should be a part of treatment initiation and continuation.

QoL should be considered an outcome of substance use treatment, as it is with any chronic illness. When paralleled with other chronic disease participants, persons with substance use disorders (SUDs) have been found to have lower mean QoL scores [34]. More specifically, the physical health and physical functioning scores were comparable to participants with chronic illness; however, due to the myriad complexities of SUD, the domains of mental health, financial status, social functioning, and productivity were markedly lower [41].

Changes in QoL may be an indicator for potential relapse and sustained remission. Although our study demonstrated a relationship between decrease in QoL and relapse, it is unclear whether relapse preceded or followed a drop in rated QoL. This study supports the body of evidence that shows an association between QoL and relapse [42,43,44], however, causation has not been established. One study examining QoL and alcohol use disorder found that improved QoL was associated with abstinence at six months, yet it could not determine if it operated as a predictor [42]. Our study demonstrated improved QoL in participants who reported continued abstinence from alcohol, supporting other research showing a positive association between QoL and abstinence [42,43,45]. Researchers examining the role of QoL satisfaction in recovery found overall satisfaction was associated with sustained abstinence, and in fact predicted remission at 1 year [43]. Another found that MOUD initiation and maintenance is imperative to improved QoL and sustained abstinence [44]. Intriguingly, a subgroup in this study who reported higher levels of substance use, legal and psychiatric problems, and inadequate family and social support did not show an improvement in quality of life related to MOUD, but instead with social support services. Our study found improvements in quality of life among participants who reported past legal problems (shoplifting or vandalism), possibly indicating the degree or severity of and/resolution of legal problems may be a factor rather than the presence of legal problems.

One of the most significant factors identified in our study related to increased QoL was attending counseling with an LCDC or professional counselor. Communicating with a health professional who is trained to listen and treat participants in a holistic manner, with empathy, has shown to create greater therapeutic engagement [46]. The link between opioid use and mental health problems has been vigorously researched and is clearly established as has a link between mental health and QoL [47,48,49], yet significant geographical and financial barriers to substance use and mental health services prevent under and uninsured participants from accessing care. Texas (located in southern United Stated) has both the largest gap between substance use treatment centers per capita and the largest uninsured population in the nation [50,51]. Novel therapies such as prescription digital therapeutics, which deliver on-demand counseling through mobile applications, may offer a solution and should be studied further [52,53,54]. As individuals with serious mental illness are less likely to have health insurance and are at greater risk of a drug overdose, these novel modalities may be funded by health systems or non-profit organizations [55,56]. Research examining mental health symptomology among illicit drug users found psychological distress significantly higher in individuals who use heroin and those who use methamphetamines than in the general population, and increasing after a non-fatal overdose [56,57]. Integrated services that include mental health support services to address psychological stressors, MOUD, and social support services, whether short-term or long-term, are essential for improving QoL.

We identified large differences between participants that started the program with a high reported QoL and those who started the program with a low QoL. As the scale is capped at a 100, participants already starting at a high QoL can improve less than those starting at low QoL, as is evident from Figure 5. It is not surprising then that the factors identified as having an associated with QoL only in the group of participants starting with high QoL are associated predominantly with a decrease in QoL from baseline. Interestingly, in the group starting with a low QoL, only the factor of relapse after joining the program remained strongly associated with QoL change, suggesting that change in QoL for this group may be governed by a combination of factors, each making a relatively small contribution. As a limitation to this observation, we note that the reduction of size of each group relative to analyzing all the participants reduces the statistical power.

Prior research has identified the prevalence between co-morbid OUD and psychiatric disorders such as bipolar disorder, anxiety disorder, and major depressive disorder, as well as suicidal ideation [58]. The objective of this research was to present sociodemographic factors that provide an alternative, patient-centric approach. However, this study had several limitations. The main limitation was the lack of a control group, which may have provided a clear evaluation of the effects of specific interventions on changes in QoL. A second limitation was the relatively small sample size, which limited the power to detect certain factors, especially when there was large variability in the QoL scores. Last, improved standardization of the QoL assessment times would provide a better view of the rate of change in QoL through the program. Selection bias may or may not account for the differences in quality of life between those who were willing to participate in the program and those who dropped out.

## 5. Conclusions

Our study examined a short-term program that offers co-located and integrated services until participants are transitioned to a long-term substance use treatment facility. Bridging programs such as the one presented in this research are an essential component of recovery, particularly in areas that lack the treatment capacity to meet the demand [50]. Quality of life is not just an important factor in long-term outcomes, but in short-term outcomes as well, as short-term outcomes influence treatment retention and continued abstinence [59]. This study shows that even brief interventions that provide a holistic approach to treatment and wellbeing, offering behavioral health interventions and social services in addition to MOUD, can help improve QoL.

## Figures and Tables

**Table 1:** Demographic characteristics of participants enrolled in a treatment program for opioid use disorder with measured QoL (*n* = 494).

Characteristic	N
Total	494 (100)
Age, mean (sd)	36.1 (9.9)
Gender †	
Male	279 (56)
Female	210 (43)
Race *	
White	428 (87)
Black or African American	60 (12)
Asian	5 (1)
Native/Hawaiian or Other Pacific Islander	4 (0.81)
Other/Did not provide	7 (1.4)
Insurance	
Commercial	68 (13.8)
Medicare	14 (2.8)
Medicaid	41 (8.3)
Uninsured/Unknown	371 (75.1)
Housing status	
Own/Rent	191 (38.7)
Live with family or friend	207 (41.9)
Homeless	49 (9.9)
Other	47 (9.5)
A veteran	14 (2.8)

Sd—standard deviation. † five participants did not disclose their gender * Some participants reported more than one race.

**Table 2:** Significant factors associated with change in QoL.

Description	BH-Adjusted *p*-Value	Mean QoL Change among Answering No	Mean QoL Change among Answering Yes
Have you relapsed since joining the program?	e^−5^	22.0	7.5
Are you currently attending LCDC or professional counseling?	0.001	5.5	19.0
Substance use history (nicotine/tobacco)	0.007	10.9	21.6
Substance use history (marijuana)	0.007	10.3	21.2
Substance use history (benzodiazepines)	0.007	11.4	21.8
Substance use history (alcohol)	0.007	10.3	21.1
Have you had any experiences you would consider to be traumatic?	0.007	6.2	25.7
Arrested for? (shoplifting or vandalism)	0.01	14.4	28.2
Status of legal problems (past)	0.02	13.2	23.6
Substance use history (non-Rx opiates)	0.03	11.5	20.6

## Data Availability

The data used in this study are available from the corresponding author upon reasonable request.
